# Studying the Effect of Taking Statins before Infection in the Severity Reduction of COVID-19 with Machine Learning

**DOI:** 10.1155/2021/9995073

**Published:** 2021-06-19

**Authors:** Alireza Davoudi, Mohsen Ahmadi, Abbas Sharifi, Roshina Hassantabar, Narges Najafi, Atefeh Tayebi, Hamideh Abbaspour Kasgari, Fatemeh Ahmadi, Marzieh Rabiee

**Affiliations:** ^1^Department of Infectious Diseases, Mazandaran University of Medical Sciences, P.O. Box: 48175-866, Sari, Iran; ^2^Antimicrobial Resistance Research Center, Communicable Diseases Institute, Mazandaran University of Medical Sciences, Sari, Iran; ^3^Department of Industrial Engineering, Urmia University of Technology (UUT), P.O. Box: 57166-419, Urmia, Iran; ^4^Department of Mechanical Engineering, Urmia University of Technology (UUT), P.O. Box: 57166-419, Urmia, Iran; ^5^Department of Clinical Pharmacy, Mazandaran University of Medical Science, P.O. Box: 48175-866, Sari, Iran

## Abstract

Statins can help COVID-19 patients' treatment because of their involvement in angiotensin-converting enzyme-2. The main objective of this study is to evaluate the impact of statins on COVID-19 severity for people who have been taking statins before COVID-19 infection. The examined research patients include people that had taken three types of statins consisting of Atorvastatin, Simvastatin, and Rosuvastatin. The case study includes 561 patients admitted to the Razi Hospital in Ghaemshahr, Iran, during February and March 2020. The illness severity was encoded based on the respiratory rate, oxygen saturation, systolic pressure, and diastolic pressure in five categories: mild, medium, severe, critical, and death. Since 69.23% of participants were in mild severity condition, the results showed the positive effect of Simvastatin on COVID-19 severity for people that take Simvastatin before being infected by the COVID-19 virus. Also, systolic pressure for this case study is 137.31, which is higher than that of the total patients. Another result of this study is that Simvastatin takers have an average of 95.77 mmHg O_2_Sat; however, the O_2_Sat is 92.42, which is medium severity for evaluating the entire case study. In the rest of this paper, we used machine learning approaches to diagnose COVID-19 patients' severity based on clinical features. Results indicated that the decision tree method could predict patients' illness severity with 87.9% accuracy. Other methods, including the *K*-nearest neighbors (KNN) algorithm, support vector machine (SVM), Naïve Bayes classifier, and discriminant analysis, showed accuracy levels of 80%, 68.8%, 61.1%, and 85.1%, respectively.

## 1. Introduction

In late December 2019, a previously unidentified coronavirus, currently named the 2019 novel *β*-coronavirus, emerged from Wuhan, China, the provincial capital of Hubei Province. The virus was later named severe acute respiratory syndrome coronavirus 2 (SARS-CoV-2) [1]. The World Health Organization (WHO) first declared the coronavirus disease (named COVID-19) as an international public health emergency and then as a pandemic [2]. The disease's incubation period is from 2 to 14 (average 4 to 7) days [1], and its initial manifestations are related to viremia. The clinical manifestations of COVID-19, which appear after an incubation period of around 5-6 days, are associated with the release of cytokines and cytokine storm syndrome in severe cases. The clinical spectrum of the disease varies from asymptomatic or mild (in more than 80%) to severe cases, which lead to acute respiratory syndrome, respiratory failure, and death. Clinical features of the disease include fever, coughing, fatigue, sweating, myalgia, sore throat, dry mouth, dry cough, shortness of breath, chest pain, hemoptysis, abdominal pain, nausea, and diarrhea [3]. According to the disease onset, the essential radiographic manifestations include scattered subpleural ground glass lesions, crazy paving lesions, and consolidation [1]. The definitive diagnosis of the disease is made by virus detection through RT-PCR. For this purpose, a sample of the pharyngeal swab, nasopharynx or oropharynx, and a sample of tracheal secretions are needed [3]. The most critical laboratory evidence of COVID-19 patients includes lymphocytopenia and increased CRP. Also, the most important risk factors include old age; diabetes; high blood pressure; chronic heart, lung, liver, and kidney diseases; cardiovascular disease; immunodeficiency; and cancers [1]. Severity criteria of the disease are SpO_2_ < 93% on room air at sea level, a respiratory rate > 30 breaths/min, PaO_2_/FiO_2_ < 300 mmHg, or lung infiltrates > 50% within 48 h [3]. Oxygen therapy, using a nasal cannula or a high-flow oxygen device, should be administered immediately. So far, there has been no conclusive evidence for the effectiveness of current antiviral therapies. In this regard, chloroquine or hydroxychloroquine and ritonavir/lopinavir (Kaletra) are used in most treatment protocols, and antiviral drugs, including Favipravir, Remdesivir, Arbidol, Sofosbuvir, and Ribavirin, are currently used in clinical trials [3].

Statins are inhibitors of the enzyme hydroxyl methylglutaryl coenzyme A (HMG-CoA reductase) and are responsible for accelerating the early stages of cholesterol biosynthesis. These compounds are multivalent cardioprotective drugs increasingly recognized as mediators with direct cellular effects beyond their cardiac role. Statins can block some downstream molecules such as farnesyl pyrophosphate (FPP) and geranylgeranyl pyrophosphate (GGPP), which play a vital role in infecting viruses like influenza. They have also been discussed in terms of intercellular, intracellular, inflammatory, and proinflammatory signals in some studies. Some research has reported their anti-inflammatory and immunomodulatory properties and upregulation for ACE2 receptors and statins [4]. It appears that lipid-lowering pharmacological interventions, in particular statins, might reduce the risk of cardiovascular complications caused by COVID-19 and might potentially have an additional antiviral activity. Several studies have shown that lipid rafts are involved in the life cycle of different viruses, including coronaviruses. Evidence of the cholesterol importance for viral entry into host cells suggests a role for cholesterol-lowering therapies in reducing viral infectivity. Statins have pleiotropic impacts, including anti-inflammatory, immunomodulatory, and antithrombotic activities, in addition to their lipid reduction and plaque stability effects. In some studies that examine statin therapy in influenza infection, lower mortality rates, and intubation, statin treatment demonstrated improved blood viral clearance throughout chronic hepatitis C infection. Statins also are used to monitor critical inhibitors of SARS-CoV-2 as a potentially SARS-CoV-2 protease [5]. Nevertheless, no proper antiviral treatment has been found for this disease so far, and all medications used are based on hypotheses that do not provide adequate evidence to support them. Due to the very high prevalence of the virus and its relatively high mortality rate, finding factors that can prevent or accelerate the onset or exacerbation of the disease and its complications can provide significant help in reducing the mortality of this disease in the current pandemic. Besides, it can be helpful for the treatment of subsequent possible seasonal epidemics such as influenza.

The present study investigates the effect of using standard doses of statins in the months before infection in patients with COVID-19 admitted to the Razi Hospital in Ghaemshahr, Iran, during February and March 2020, to reduce the severity of the disease and mortality rate of COVID-19. Overall, using statins may be a good guideline in the initial months of the COVID-19 epidemic.

## 2. Literature Review

Virani [6] conducted a review study to assess whether ongoing statin therapy enhances the overall cardiovascular outcomes of virally infected patients, like COVID-19. According to this paper, none of the studies reported adverse effects of this therapy. Fedson et al. [7] indicated the positive effects of statin adjuvant therapy in Sierra Leone in the 2014 outbreak of Ebola treatments. Zhang et al. [8] assessed the risk of entering COVID-19 with the decrease in ACE2 expression. A retrospective analysis was presented in 13,981 COVID-19 patients, including 1219 statins, in Hubei Province, China. Based on a mixed-effect Cox model, after the tendency match, the probability of all-cause death for 28 days was 5.2% and 9.4%, with an adjusted hazard ratio of 0.58 for both the matched statin and nonstatin classes. The lower mortality risks involved with statin use were recorded in Cox's time-varying method and marginal structural model study. The possible significant improvements of statins on COVID-19 patients were addressed by Rodrigues-Diez et al. [9]. Overall, they could target infected cell virus receivers, replications, degrading, and downstream reactions by discussing central and epidemiological proof. According to their results, statins might modulate virus entrance, acting on the SARS-CoV-2 receptors, ACE2 and CD147, and the involvement of lipid rafts. Besides, statins may control viral replication or degradation and have protective effects by inducing autophagic activation.

By closing multiple molecular pathways, including NF-*κ*B and NLRP3 inflammasomes, the well-known anti-inflammatory effects of statins might restrict the cytokine storm in extreme COVID-19 patients associated with fatal outcomes. In conclusion, statin moderation of stimulation of coagulation reaction can also help boost the results of COVID-19. According to Castiglione et al. [10], statins are low-cost, widely tested, and well-tolerated medicines. These compounds are less likely to be affected due to health emergencies such as the ongoing COVID-19 pandemic, including in low-income countries, where therapy with costly medicines is not feasible. Adjuvant therapy and further treatment of preestablished statins might enhance the clinical success of COVID-19 patients through either immunomodulatory behavior or cardiovascular damage prevention. Subir et al. [11] have shown that statin can minimize the seriousness of lung injury and mortality from extreme acute respiratory syndrome-coronavirus 2 (SARS-CoV2) infections because of its immunomodulatory, anti-inflammatory, antithrombotic, and antioxidant properties. Upregulation of statin-induced angiotensin-converting enzyme-2 (ACE2) can also minimize lung damage due to excess angiotensin II. Statins can reduce viral entry into cells by disturbing lipid rafts. Daniels et al. [12] examined the relationship using statin/angiotensin-converting enzyme inhibitors/ARB in patients hospitalized for COVID-19 in the month before hospitalization. This study incorporated factors such as the risk of the severe result and time for the extreme outcome or disease treatment. They show that obesity and diabetes are potentially severe consequences of COVID-19.

Further, the predicted effects of the male sex consistently lead to an increased risk. A relationship was obtained between COVID status and obesity in COVID-negative and COVID-positive patients as a protective and risk factor. One new research in this regard shows that a shorter recovery period is associated with a younger generation. It may represent a more robust population and the fact that younger individuals subsequently present disease over time. While current smoking was more prominent in moderate rather than serious COVID-19, this cohort was questionable for its validity due to the very low prevalence of smoking (only eight current smokers have been found). Reiner et al. [13] suggested that statins could be effective inhibitors of SARS-CoV-2 M pro, based on binding energy from pitavastatin, Rosuvastatin, Lovastatin, and Fluvastatin. This claim is supported by the fact that certain statins (especially pitavastatin) have an even more considerable binding energy than protease or polymerase inhibitors.

## 3. Methods and Materials

### 3.1. Mechanism of Statin Action with COVID-19

The primary way of COVID-19 virus infection in body cells is ACE2, which downregulates this enzyme in the cells and lowers its protection properties. The virus triggers the response of the proinflammatory host based on MYD88, TLR, and NF-*κ*B pathway activations. Statins are widely accessible, inexpensive, healthy, fat-reducing, and immunomodulatory medicines. These compounds prevent proinflammation of the MYD88-NF-*μ*B and facilitate the upregulation of ACE2 in experimental models. Statins can be effective in the treatment of COVID-19 patients through these pathways. Statins also counteract hyperlipidemia triggered by some therapies commonly used in antiviral and immunosuppressive COVID-19 [10].

Like avian influenza viruses, by causing an extreme proinflammatory host reaction, beta-coronaviruses cause serious respiratory diseases. Some immunomodulatory treatments have proven to be successful in SARS, MERS, and COVID-19 cases. For instance, tocilizumab, an anti-interleukin-6 receptor humanized monoclonal antibody, was beneficial as maintenance care in selected patients with COVID-19 [14]. The interaction of SARS-CoV-1 with Toll-like receptors on the host cell membrane dramatically enhances the activity of the gene MYD88, whose output stimulates the occurrence of NF-*κ*B-causing inflammatory processes [15]. In a murine model of SARS-CoV-1 infection, inhibition of NF-*κ*B caused a reduction in lung infection and improved the survival rate of the disease [16]. Observational models suggest that statins stabilize MYD88 following a proinflammatory stimulus, including hypoxia [17]. Also, NF-*κ*B activation was significantly decreased within 48 h in murine cells (relating to the plasma levels obtained with a healthy human dose of 40 mg [18]). Based on this information, the use of statins can be considered an immunomodulatory treatment in patients with COVID-19.

**Figure 1:**
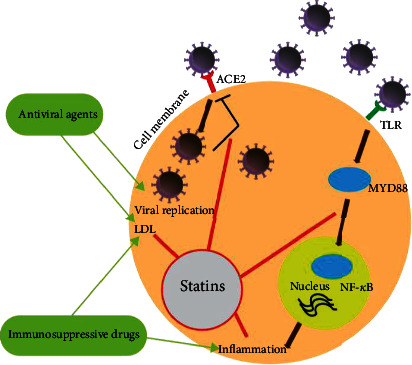
Statin action against COVID-19 virus.

Statins also interrupt the signaling of ACE2. After initial entry via ACE2, SARS-CoV-2 downregulates the expression of ACE2. As a result, it may foster original infiltration by innate immune cells and trigger an uncontested accumulation of angiotensin II, injuring the organ [14]. Both statins and ARBs are considered epigenetic modifications to regulate ACE2 (Figure 1) [7] experimentally. Regarding the improving effects of ACE2 on COVID-19 patients, there are currently activated RCTs with recombinant human ACE2 or ARBs1, and biological plausibility is also present in the study of statins [7].

### 3.2. Clinical Criteria and Variables

Indications for COVID-19 hospitalization are respiratory rate (RR) > 24, oxygen saturation (O_2_Sat) < 93, significant lesion on CXR CT scan, pulmonary infiltration, and clinical judgment of a physician [9]. Criteria for severe disease include the number of breaths ≥ 30 times per minute, arterial oxygen saturation < 93 when the patient breathes in room air, and severe multifocal pulmonary involvement increases by more than 50% within 48 h [3].

## 4. Results and Discussion

### 4.1. Gathering Data

The present study investigates the effect of using standard doses of statins in the months before infection in patients with COVID-19 admitted to the Razi Hospital in Ghaemshahr (Mazandaran Province, Iran) during February and March 2020 in reducing the severity of the disease and mortality rate of COVID-19. The recorded variables and patients are illustrated in Table 1.

This study investigates whether the severity of COVID-19 disease differs from patients who have previously taken statins due to hyperlipidemia or cardiovascular disease compared to patients who did not take statins before. In other words, the main objective is to explore if the history of taking statins has a positive effect on the COVID-19 disease process. It is of note that during the study, the patients did not use any statin during hospitalization. Table 1 shows the descriptive statistics of the patients who participated in this clinical research. The demographic data consist of age and gender, which were encoded to numerical values. Also, other criteria are past medical history; underlying diseases such as diabetes, hypertension, heart failure, chronic kidney disease (CKD), and chronic liver disease; history of transplantation; ischemic heart disease; dyslipidemia; thalassemia major; allergic asthma; hypothyroidism; history of radiotherapy; history of chemotherapy; solid organ involvement in cancer; bone marrow involvement; history of contact with COVID-19 patients; hemodialysis; and other underlying diseases such as favism, rheumatoid arthritis (RA), asthma, and stroke. Other variables are the history of steroid treatment, steroid dose, and history of addiction or smoking.

**Figure 2:**
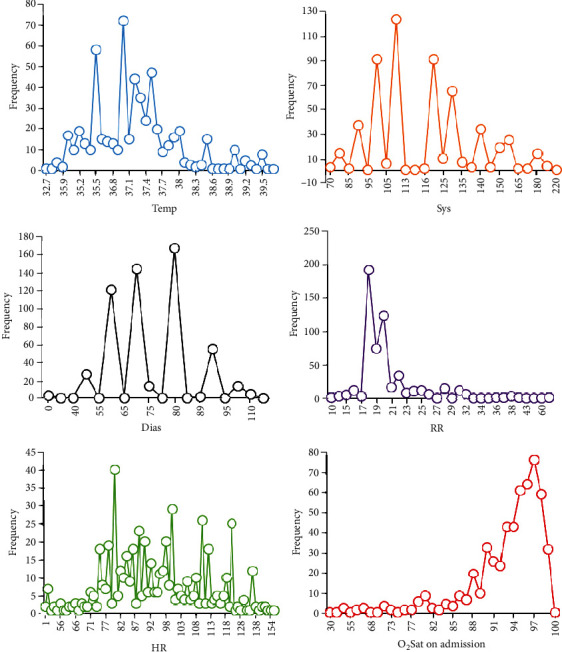
Frequency statistic vital signs of COVID-19 patients in admission.

The mentioned features were encoded binary. These encoded features, along with other features, are presented in Table 1. Clinical signs for which the patient has referred to the hospital include fever, chills, rhinorrhea, dry cough, productive cough, weakness, anorexia, sweating, headaches, myalgia, loss of taste, anosmia, hematemesis, diarrhea, stomachache, epigastric pain, dizziness, throat itching, nausea, vomiting, shortness of breathing, dyspnea, tachypnea, wheezing, chest pain, fatigue, heart palpitations, chest tightness, and sore throat. Moreover, vital signs of the patient include body temperature (Temp), systolic pressure (Sys), diastolic pressure (Dias), respiratory rate (RR), heart rate (HR), and oxygen saturation (O_2_Sat).  Figure 2 shows the frequency statistics of vital signs of all COVID-19 patients participating in this research. Based on the results, some of the patients have fever temperatures between 36.4 and 37.7°C. Also, systolic pressure for all of the patients is between 90 and 130 mmHg. The respiratory rate for most patients is in the range of 17-21 Br/min, which is not in tachypnea condition. Regarding oxygen saturation as the essential factor of COVID-19 severity, its value is between 80 and 100 mmHg for most target patients.

### 4.2. Investigation of Effects of Statins on COVID-19 Severity

The most critical factor in this study is calculating the COVID-19 severity in numerical analysis. Based on the clinical sign of the patients, we encode the seriousness as follows:

(1) $$\begin{array}{c} \left\{\begin{array}{cc} 1\text{ else} & \text{Mild},\\ 2\;90\%<{\text{O}}_{2}\text{Sat}\leq 93\% & \text{Medium},\\ 3\;88\%<{\text{O}}_{2}\text{Sat}\leq 90\%,\text{RR}>30 & \text{Severe},\\ 4\;{\text{O}}_{2}\text{Sat}\leq 88\%,\text{Sys}<90,\text{Dias}<60\text{ mmHg} & \text{Critical},\\ 5\text{ Death} & \text{Death}. \end{array}\right. \end{array}$$

This study tried to evaluate the severity of all patients according to the history of statin taking of the patients, and the obtained results are described in Table 2. The examined research patients include people that had taken three types of statins consisting of Atorvastatin, Simvastatin, and Rosuvastatin. Of 561 patients, 17.3%, 2.3%, and 0.5% of them have used Atorvastatin, Simvastatin, and Rosuvastatin statins, respectively, for past disease treatment. Of all people who take Atorvastatin, 37.1% have mild COVID-19, and 25.8% have critical conditions. For Simvastatin, 69.23% of the patients have mild COVID-19. However, most people taking Rosuvastatin are in a critical situation.

One method for evaluating the relationship between statins' effects and severity is the statistical correlation test.  Table 3 presents the correlation test findings based on Spearman's methods. The results show an indirect relationship between taking Simvastatin and severity. Regarding these findings, people who have taken Simvastatin are of lower severity than others. Moreover, most of them (69%) had mild severity. On the other hand, most patients who take Rosuvastatin are in critical condition. Furthermore, there is no significant relationship between Atorvastatin users and COVID-19 severity.

According to the results, Simvastatin reduced COVID-19 severity significantly. In Table 4, these people have been evaluated based on vital signs. For the entire case study, the average fever temperature of patients is 37.2°C. However, the number of Simvastatin users is 36.831, which is lower than the total number of patients (i.e., 372°C). Moreover, systolic pressure for this case study is 137.31, which is higher than that of total patients.

Based on the obtained results, diastolic pressure for both groups is almost equal. Also, the heart rate for Simvastatin takers is lower than the entire case study, and the respiratory rate is high in Simvastatin takers. The most critical parameter of patients for this comparison is oxygen saturation. In this respect, Simvastatin takers have a 95.77 mmHg O_2_Sat, which puts them in the mild group. However, in evaluating the complete case study, the O_2_Sat is 92.42, putting them in the category of patients with medium severity.

**Figure 3:**
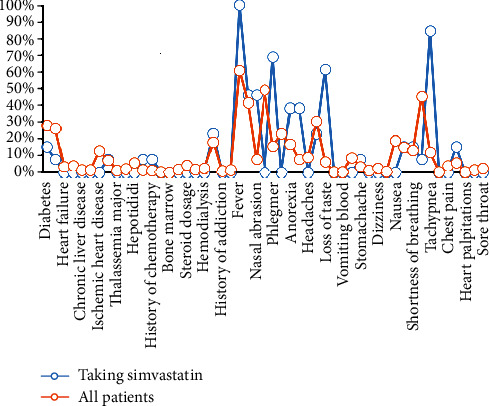
Study of patients taking Simvastatin and comparison with the total case study based on clinical symptoms.

In conclusion, we can estimate the positive influence of Simvastatin on COVID-19 severity for people that take Simvastatin before infection to the COVID-19 virus. The results of studying clinical symptoms are illustrated in Figure 3. The vertical axis shows the percentage of people with particular symptoms or historical illnesses for both case studies. Based on these results, 28% of all patients have diabetes, while only 15.38% of Simvastatin takers are involved in diabetes. Moreover, 61.14% of all patients have a fever in admission, while 100% of Simvastatin takers have a fever. None of the patients who have taken Simvastatin statin had a dry cough, while 49.20% (almost half) showed dry cough symptoms. In addition, no one has weakness, headache, anosmia, vomiting blood, diarrhea, epigastric pain, dizziness, throat itching, nausea, wheezing, chest pain, heart palpitations, chest tightness, and sore throat, among Simvastatin takers.

The significant signs of this subgroup are tachypnea or respiratory rate higher than 20 breaths per minute, given that 84.62% have tachypnea. Besides, 61.54% of Simvastatin takers lost their taste ability. Moreover, 69.23% of this case study has a productive cough in admission.

### 4.3. Diagnosis of COVID-19 Severity Based on Machine Learning Methods

Computer-aided diagnosis (CAD) tools have been recently used to study various features' impact and identify various diseases from the patient data [19]. Computationally efficient artificial neural networks (ANNs) [20, 21] have been utilized to monitor the patients' health status and diagnose various diseases such as COVID-19 and mental health disorders [21] using smartphones and smartwatches. Machine learning methods, particularly ANNs, have also been used on lung X-ray images to detect COVID-19 in the lung tissue and detect the infected areas.

**Figure 4:**
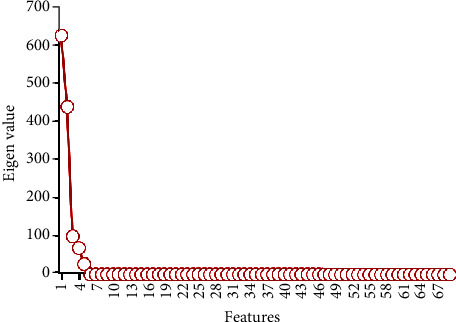
Results of feature reduction using PCA.

Inspired by machine learning applications in intelligent healthcare and investigating various aspects of COVID-19 disease, we designed machine learning networks to diagnose the severity of COVID-19 patients based on the variables (features) mentioned before. In this regard, initially, there are 69 features as independent variables. However, to obtain the best and uncomplex nonparametric classification, we should reduce this number. Therefore, principal component analysis (PCA) was used to reduce the number of initial features. The results of the PCA method are shown in Figure 4. Based on eigenvalues resulting from PCA, the number of features is reduced to 5, suggesting that we should use five features to classify and diagnose the patients' severity.

Besides, the severity factor consists of categorical labels from 1 to 5 according to Equation (1). It is also assigned as a dependent variable for diagnosis. Here, it is aimed to find machine learning architectures to diagnose COVID-19 patients' severity based on clinical signs.

**Figure 5:**
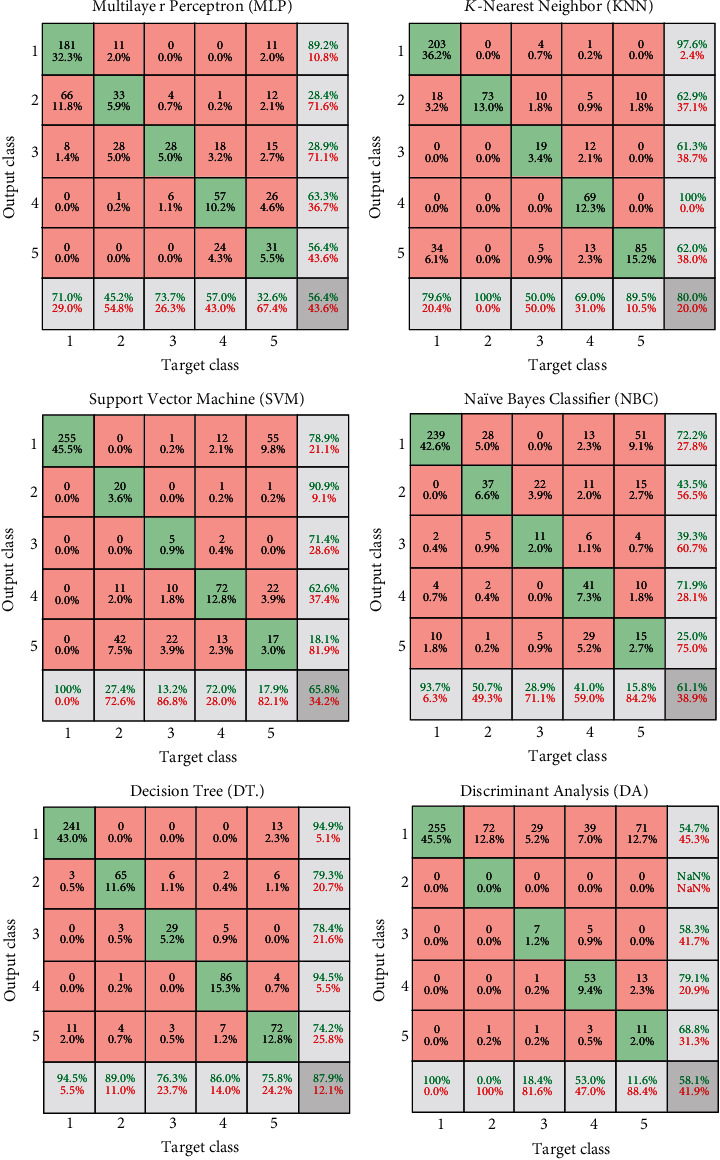
Confusion matrixes of classification using machine learning approaches.

To diagnose the patients' severity, we used six types of machine learning classifiers, including multilayer perceptron (MLP), *K*-nearest neighbors (KNN), support vector machine (SVM), Naïve Bayes classifier (NBC), decision tree (DT), and discriminant analysis (DA). The confusion matrixes of the classification methods are illustrated in Figure 5. These matrixes consist of 5 × 5 class matrixes (red) that its orthogonal elements are true values (green), and red elements are false detection values. In these matrixes, gray elements show the method's sensitivity (horizontal) and precision (vertical).

The low corner element indicates the classification accuracy. For example, in Figure 5, at MLP matrix, from 255 patients with mild severity condition, 181 are diagnosed correctly. In other words, the sensitivity for this class is 71%. However, 66 of them are diagnosed as medium severity. The MLP architecture consists of three hidden layers with 20, 10, and 1 neuron(s), in the order of their appearance. The absolute accuracy for the MLP approach is 58.8% (41.2% loss). In the DT method, the final accuracy is 87.9%, which is higher than that of other KNN, SVM, NBC, and DA (i.e., 80%, 68.8%, 61.1%, and 85.1%, respectively). In the DT method, the highest sensitivity belongs to mild patients. In other words, 94.5% of mild patients are diagnosed correctly. Regarding other severity groups, the sensitivity is 89%, 76.3%, 86%, and 75.8% for medium, severe, critical, and death people, respectively. Finally, it can be concluded that the DT methods are the best classifier among machine learning methods for diagnosing COVID-19 patients from clinical features.

## 5. Limitations

In this study, medical information may face limitations that can prevent some of the use or disclosure. For example, there are certain restrictions on using specific categories of information (i.e., HIV testing or treatment of mental illness). Also, government medical insurances restrict the disclosure of beneficiary information for purposes not related to these insurances. These limitations have made it very difficult to access all COVID-19 patient information in this study. To deal with this shortcoming, we chose patients with complete health information. The other limitation is the lack of personal information from the patient to our specialist doctors.

## 6. Conclusion

Statins are multivalent cardioprotective drugs increasingly recognized as mediators with direct cellular effects beyond their cardiac role. These drugs inhibit the enzyme hydroxyl methylglutaryl coenzyme A (HMG-CoA reductase) and are responsible for accelerating the early stages of cholesterol biosynthesis. In this study, the role and possible anti-inflammatory effects of this drug are investigated. Statins that are commonly prescribed in Iran include Atorvastatin and Simvastatin. This investigation is a retrospective descriptive-analytical cross-sectional study based on the medical records of patients. According to the preliminary information of the project implementers, more than 1500 patients with COVID-19 have been hospitalized at this center from February and March 2020. In this study, the medical records of the patients were examined. Next, their clinical and laboratory characteristics, including the history of taking statins before the onset of the disease, were entered into a previously prepared and reproduced form of information. Only patients who make a definitive diagnosis based on virus isolation by RT-PCR with a swab of the throat, nasopharynx, or oropharynx and a sample of tracheal secretions or typical radiological findings were included. Severity criteria include the number of breaths equal to or more than 30 beats per minute, arterial oxygen saturation less than 93 (when the patient breathes in-room air), severe multifocal pulmonary involvement (which increases by more than 50% within 48 h), and the need for intubation and mechanical ventilation, CPAP, and BIPAP. This paper evaluated the effects of statin taking before infection on COVID-19 severity. Moreover, machine learning methods were used to diagnose COVID-19 severity based on clinical features. Overall, the results can be summarized as follows:

- There is an indirect (positive) relationship between taking Simvastatin and COVID-19 severity
- People who have taken Simvastatin are of lower severity than others
- About 69% of Simvastatin takers are of mild severity
- There is no significant relationship between Atorvastatin users and COVID-19 severity
- Most patients who take Rosuvastatin are in critical condition
- The average fever temperature of all case studies is 37.2°C
- The average fever temperature of Simvastatin takers is 36.8°C
- The systolic pressure for Simvastatin takers is 137.31 mmHg
- The heart rate for Simvastatin takers is lower than the entire case study
- The respiratory rate is high in Simvastatin takers
- Simvastatin takers have a 95.77 mmHg oxygen saturation, placing them in mild severity conditions
- The average oxygen saturation of all case studies is 92.42 mmHg, which puts them in mild severity conditions
- About 84.62% of Simvastatin takers have tachypnea
- About 61.54% of Simvastatin takers lost their taste ability
- Principle component analysis (PCA) was used to reduce initial features from 71 to 5
- The accuracy of the decision tree method is 87.9%, which is higher than that of other approaches
- The accuracy of KNN, SVM, NBC, and DA is 80%, 68.8%, 61.1%, and 85.1%, respectively
- The sensitivity of the DT method for patient diagnosis is 89%, 76.3%, 86%, and 75.8% for medium, severe, critical, and dead people, respectively

In conclusion, we can estimate the positive influence of Simvastatin on COVID-19 severity for people that take Simvastatin before infection to the COVID-19 virus. Furthermore, it was found that the decision tree method is an effective tool to predict the patients' severity based on clinical symptoms.

## Figures and Tables

**Table 1:** Data descriptive analysis.

	*N*	Minimum	Maximum	Mean	Std. deviation	Unit
Age						
<50 = 1	561	1	3	1.88	0.821	Year group
50‐65 = 2						
>65 = 3						
Gender						
F = 1	561	1	2	1.56	0.497	M/F
M = 2						
Duration of hospitalization	561	0	24	5.51	3.823	Days
Death	561	0	1	0.17	0.375	+/-
Diabetes	561	0	1	0.28	0.449	+/-
Hypertension	561	0	1	0.26	0.440	+/-
Heart failure	561	0	1	0.03	0.176	+/-
Chronic kidney disease	561	0	1	0.04	0.194	+/-
Chronic liver disease	561	0	1	0.01	0.119	+/-
History of transplantation						
Solid‐organ transplant = 1	561	0	1	0.01	0.119	+/-
Hematological = 2						
Ischemic heart disease	561	0	1	0.13	0.335	+/-
Dyslipidemia	561	0	1	0.07	0.261	+/-
Thalassemia major	561	0	1	0.01	0.103	+/-
Allergic asthma	561	0	1	0.02	0.145	+/-
Hepatoid	561	0	1	0.06	0.232	+/-
History of radiotherapy	561	0	1	0.01	0.119	+/-
History of chemotherapy	561	0	1	0.01	0.103	+/-
Solid organs	561	0	1	0.00	0.060	+/-
Bone marrow	561	0	0	0.00	0.000	+/-
Steroid therapy	561	0	1	0.02	0.132	+/-
Steroid dosage						
>20 mil = 1	561	0	5	0.04	0.346	Mil
>20 mil = 2						
Prednisolone total > 300 = 3						
Contact history	561	0	1	0.02	0.132	+/-
Hemodialysis	561	0	1	0.02	0.151	+/-
Another underlying disease	561	0	1	0.18	0.386	+/-
Atorvastatin	560	0	1	0.17	0.379	+/-
Simvastatin	561	0	1	0.02	0.151	+/-
Rosuvastatin	561	0	1	0.01	0.073	+/-
Binary statin (statin or not)	561	0	1	0.18	0.388	+/-
History of addiction	561	0	1	0.01	0.084	+/-
Smokers	561	0	1	0.02	0.126	+/-
Fever	561	0	1	0.61	0.488	+/-
Chills	561	0	1	0.42	0.494	+/-
Rhinorrhea	561	0	1	0.08	0.266	+/-
Dry cough	561	0	1	0.49	0.500	+/-
Productive cough	561	0	1	0.16	0.362	+/-
Weakness	561	0	1	0.23	0.422	+/-
Anorexia	561	0	1	0.17	0.375	+/-
Sweating	561	0	1	0.08	0.269	+/-
Headaches	561	0	1	0.09	0.288	+/-
Myalgia	561	0	1	0.30	0.461	+/-
Loss of taste	561	0	1	0.06	0.245	+/-
Anosmia	561	0	1	0.00	0.042	+/-
Hematemesis	561	0	1	0.00	0.042	+/-
Diarrhea	561	0	1	0.09	0.283	+/-
Stomachache	561	0	1	0.03	0.181	+/-
Epigastric pain	561	0	1	0.01	0.103	+/-
Dizziness	561	0	1	0.02	0.156	+/-
Throat itching	561	0	1	0.01	0.073	+/-
Nausea	561	0	1	0.19	0.393	+/-
Vomiting	561	0	1	0.15	0.359	+/-
Shortness of breathing	561	0	1	0.13	0.341	+/-
Dyspnea	561	0	1	0.45	0.498	+/-
Tachypnea	561	0	1	0.12	0.325	+/-
Wheezing	561	0	1	0.00	0.060	+/-
Chest pain	561	0	1	0.04	0.190	+/-
Fatigue	561	0	1	0.06	0.232	+/-
Heart palpitations	561	0	1	0.00	0.042	+/-
Chest tightness	561	0	1	0.02	0.126	+/-
Sore throat	561	0	1	0.02	0.156	+/-
Temp	561	32.7	39.7	37.202	0.8102	°C
Sys	561	70	220	119.22	22.879	mmHg
Dias	561	0	120	72.59	13.838	mmHg
RR	561	10	75	20.76	5.682	Br/min
HR	561	1.0	170.0	93.766	21.0701	BPM
O_2_Sat on admission	561	30	100	92.42	8.117	mmHg
CT scan						
gloss opacity and increase in thickness between lobules or inside=1	561	0	7	0.32	0.920	0-7
Multiple alveolar consolidation = 2A						
Alveolar consolidation local = 2B						
Reversed halo = 3						
Bronchovascular thickening in the lesion = 4						
Tractional bronchiectasis = 5						
Fiber tapes = 6						
Acute respiratory distress syndrome = 7						
Intensive cares						
1 = primary hospitalization in ICU	561	0	2	0.32	0.686	0-2
2 = transfer from another part to ICU						
Noninvasive ventilation						
Nasal O_2_ = 1	561	0	4	0.30	0.763	0-4
Mask O_2_ = 2						
CPAP = 3						
BIPAP = 4						
Mechanical ventilation	561	0	2	0.14	0.350	0-2
Vasopressor						
Norepinephrine = 1	561	0	3	0.08	0.394	0-3
Dopamine = 2						
Dobutamine = 3						
Severity	561	1	5	2.48	1.592	1-5

**Table 2:** Description statistic of disease severity of the patients that take statins of Atorvastatin, Simvastatin, and Rosuvastatin.

			Severity					Total
			1	2	3	4	5	
Atorvastatin	0	Count	219	60	29	75	80	463
		% of total	47.30%	12.96%	6.26%	16.20%	17.28%	82.7%
	1	Count	36	12	9	25	15	**97**
		% of total	**37.1%**	**12.4%**	**9.3%**	**25.8%**	**15.4%**	**17.3%**

Simvastatin	0	Count	246	72	37	99	94	548
		% of total	44.89%	13.14%	6.75%	18.07%	17.15%	97.7%
	1	Count	9	1	1	1	1	**13**
		% of total	**69.23%**	**7.69%**	**7.69%**	**7.69%**	**7.69%**	**2.3%**

Rosuvastatin	0	Count	255	73	38	98	94	558
		% of total	45.70%	13.08%	6.81%	17.56%	16.85%	99.5%
	1	Count	0	0	0	2	1	**3**
		% of total	**0.00%**	**0.00%**	**0.00%**	**66.67%**	**33.33%**	**0.5%**

Total		Count	255	73	38	100	95	561
		% of total	45.5%	13.0%	6.8%	17.8%	16.9%	100.0%

**Table 3:** Results of Spearman correlation for the effects of statin history on COVID-19 severity.

		Atorvastatin	Simvastatin	Rosuvastatin	Severity
Atorvastatin	Correlation coefficient Sig. (1-tailed)	1.000	-0.071^∗^ (0.048)	-0.034 (0.214)	0.065 (0.063)
Simvastatin	Correlation coefficient Sig. (1-tailed)		1.000	-0.011 (0.395)	**-0.072** ^∗^ **(0.044)**
Rosuvastatin	Correlation coefficient Sig. (1-tailed)			1.000	**0.080** ^∗^ **(0.028)**
Severity	Correlation coefficient Sig. (1-tailed)				1.000

**Table 4:** Study of patients taking Simvastatin and comparison with the total case study based on vital signs.

	Taking Simvastatin			All patients		
	*N*	Mean	Std. deviation	No.	Mean	Std. deviation
Temp	13	36.831	0.6033	561	37.202	0.8102
Sys	13	137.31	25.869	561	119.22	22.879
Dias	13	75.31	25.650	561	72.59	13.838
RR	13	21.92	6.538	561	20.76	5.682
HR	13	91.77	16.233	561	93.766	21.0701
O_2_Sat	13	95.77	2.127	561	92.42	8.117
Severity	13	1.77	1.363	561	2.48	1.592

## Data Availability

The present study investigates the effect of using standard doses of statins in the months before infection in patients with COVID-19 admitted to Razi Hospital in Ghaemshahr (Mazandaran Province, Iran), and the data of the article is unpublishable due to the preservation of patients' information.

## References

[1] Guan W., Ni Z. Y., Hu Y. (2020). Clinical characteristics of coronavirus disease 2019 in China. *The New England Journal of Medicine*.

[2] World Health Organization (2020). *Events as they happen*.

[3] Li T. (2020). Diagnosis and clinical management of severe acute respiratory syndrome Coronavirus 2 (SARS-CoV-2) infection: an operational recommendation of Peking Union Medical College Hospital (V2.0): Working Group of 2019 Novel Coronavirus Peking Union Medical College Hospital. *Emerging Microbes & Infections*.

[4] Mehrbod P., Omar A. R., Hair-Bejo M., Haghani A., Ideris A. (2014). Mechanisms of action and efficacy of statins against influenza. *BioMed Research International*.

[5] Radenkovic D., Chawla S., Pirro M., Sahebkar A., Banach M. (2020). Cholesterol in relation to COVID-19: should we care about it?. *Journal of Clinical Medicine*.

[6] Virani S. S. (2020). *Is there a role for statin therapy in acute viral infections?*.

[7] Fedson D. S., Opal S. M., Rordam O. M. (2020). Hiding in plain sight: an approach to treating patients with severe covid-19 infection. *MBio*.

[8] Zhang X.-J., Qin J. J., Cheng X. (2020). In-hospital use of statins is associated with a reduced risk of mortality among individuals with COVID-19. *Cell Metabolism*.

[9] Rodrigues-Diez R. R., Tejera-Muñoz A., Marquez-Exposito L. (2020). Statins: could an old friend help in the fight against COVID-19?. *British Journal of Pharmacology*.

[10] Castiglione V., Chiriacò M., Emdin M., Taddei S., Vergaro G. (2020). Statin therapy in COVID-19 infection. *European Heart Journal - Cardiovascular Pharmacotherapy*.

[11] Subir R., Mukherjee Jagat J., Gangopadhyay Kalyan K. (2020). Pros and cons for use of statins in people with coronavirus disease-19 (COVID-19). *Diabetes and Metabolic Syndrome: Clinical Research & Reviews*.

[12] Daniels L. B., Sitapati A. M., Zhang J. (2020). Relation of statin use prior to admission to severity and recovery among COVID-19 inpatients. *The American Journal of Cardiology*.

[13] Reiner Ž., Hatamipour M., Banach M. (2020). Statins and the Covid-19 main protease: in silico evidence on direct interaction. *Archives of Medical Science*.

[14] Madjid M., Safavi-Naeini P., Solomon S. D., Vardeny O. (2020). Potential effects of coronaviruses on the cardiovascular system: a review. *JAMA Cardiology*.

[15] Totura A. L., Whitmore A., Agnihothram S. (2015). Toll-like receptor 3 signaling via TRIF contributes to a protective innate immune response to severe acute respiratory syndrome coronavirus infection. *MBio*.

[16] DeDiego M. L., Nieto-Torres J. L., Regla-Nava J. A. (2014). Inhibition of NF-*κ*B-Mediated inflammation in severe acute respiratory syndrome coronavirus-infected mice increases survival. *Journal of Virology*.

[17] Yuan X., Deng Y., Guo X., Shang J., Zhu D., Liu H. (2014). Atorvastatin attenuates myocardial remodeling induced by chronic intermittent hypoxia in rats: partly involvement of TLR-4/MYD88 pathway. *Biochemical and Biophysical Research Communications*.

[18] Chansrichavala P., Chantharaksri U., Sritara P., Chaiyaroj S. C. (2009). Atorvastatin attenuates TLR4-mediated NF-*κ*B activation in a MyD88-dependent pathway. *Asian Pacific Journal of Allergy and Immunology*.

[19] Ahmadi M., Sharifi A., Hassantabar S., Enayati S. (2021). QAIS-DSNN: tumor area segmentation of MRI image with optimized quantum matched-filter technique and deep spiking neural network. *BioMed Research International*.

[20] Hassantabar S., Stefano N., Ghanakota V. (2020). Coviddeep: Sars-cov-2/covid-19 test based on wearable medical sensors and efficient neural networks. http://arxiv.org/abs/2007.10497.

[21] Hassantabar S., Ahmadi M., Sharifi A. (2020). Diagnosis and detection of infected tissue of COVID-19 patients based on lung X-ray image using convolutional neural network approaches. *Chaos, Solitons & Fractals*.

